# Supplementary material for: Lifespan regulation by targeting heme signaling in yeast
Source: GeroScience. 2024 May 29;46(5):5235–45. doi: 10.1007/s11357-024-01218-9 (PMC11335709; doi:10.1007/s11357-024-01218-9)
Supplement: Supplementary file 1 — Supplementary file1 (PDF 799 KB) [file 11357_2024_1218_MOESM1_ESM.pdf]

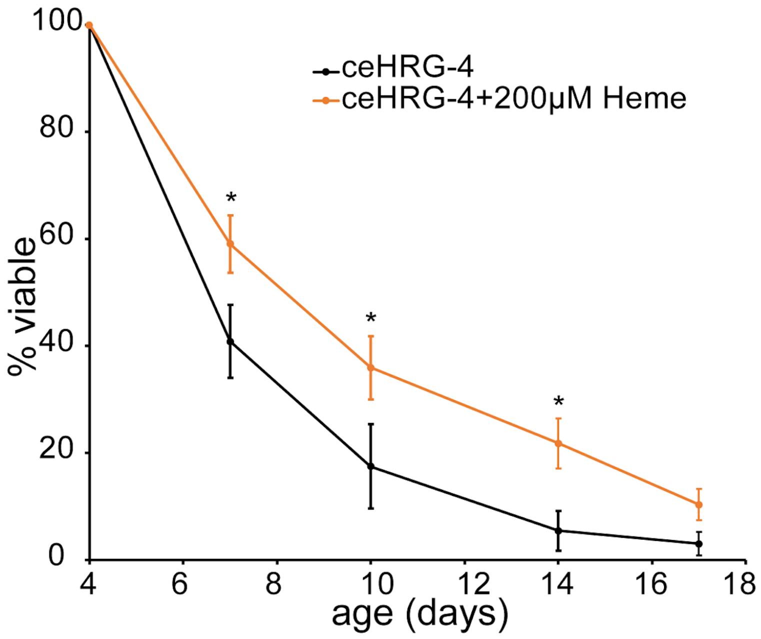

Supplementary Figure S1. Heme supplementation extends chronological lifespan of ceHRG-4 expressing cells. Error bars represent SEM of six independent experiments. *, $p<0.05$ (two-way ANOVA).

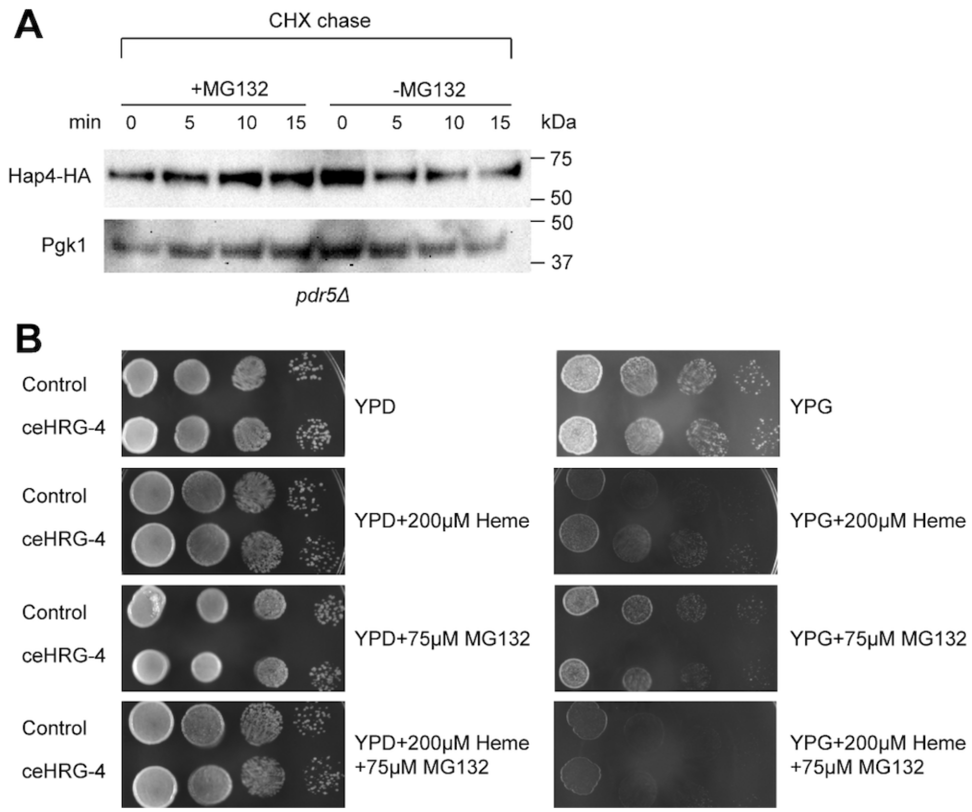

Supplementary Figure S2. Heme supplementation does not affect the proteasomal degradation of Hap4. **A**) Degradation of Hap4 is prevented in the presence of proteasome inhibitor. Cycloheximide (CHX) chase assay was performed in *pdr5Δ* cells in the presence or absence 75 µM MG132 proteasome inhibitor. Hap4-HA protein levels were analyzed by Western blotting with an anti-HA antibody. Pgk1 was used as a loading control. Representative results from three independent experiments are shown. **B**) The proteasomal inhibitor does not rescue the growth of ceHRG-4 expressing cells treated with heme on glycerol-containing medium (YPG). Yeast strains were cultured in YPD media overnight and serial (10x) dilutions were spotted on YPD and YPG plates containing 200 µM heme, 75 µM MG132, or both. Plates were incubated at 30°C for 2 days prior to imaging.

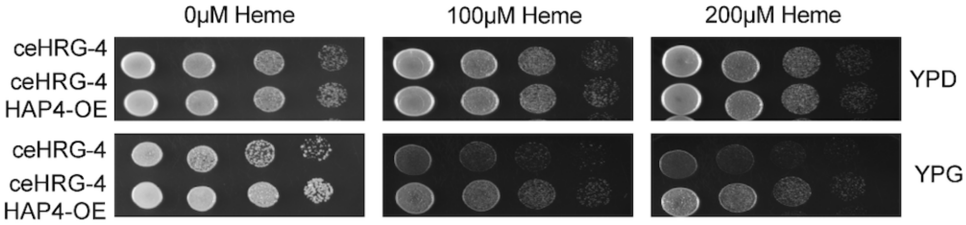

Supplementary Figure S3. *HAP4* overexpression (HAP4-OE) rescues the growth of ceHRG-4 expressing cells supplemented with heme on glycerol containing medium (YPG). Representative images from two independent experiments are shown.

Supplementary Table S1. Yeast strains used in this study.

Identifier	Genotype	Source
PP109 (BY4741)	*MATa his3Δ1 leu2Δ0 met15Δ0 ura3Δ0*	Horizon Discovery
PP408	*MATa his3Δ1 leu2Δ0 met15Δ0 ura3Δ0 hmx1Δ::ADH1pr-HMX1-URA3*	This study
PP438	*MATa his3Δ1 leu2Δ0 met15Δ0 ura3Δ0 hem1Δ::KanMX4*	This study
PP454	*MATa his3Δ1 leu2Δ0 met15Δ0 hem1Δ::KanMX4 ura3Δ::pGAP-ceHRG-4-HA-URA3*	This study
PP473	*MATa his3Δ1 leu2Δ0 met15Δ0 ura3Δ::GPDpr-ceHRG-4-HA-URA3*	This study
PP488	*MATa his3Δ1 leu2Δ0 met15Δ0 hap4Δ::HAP4-HA-KanMX4 ura3Δ::GPDpr-ceHRG-4-HA-URA3*	This study
PP499	*MATa his3Δ1 leu2Δ0 met15Δ0 ura3Δ::GPDpr-ceHRG-4-HA-KanMX4 hap4::ADH1pr-HAP4-URA3*	This study
PP505	*MATa his3Δ1 leu2Δ0 met15Δ0 ura3Δ0 hap4Δ::KanMX4*	This study
PP553	*MATa his3Δ1 leu2Δ0 met15Δ0 ura3Δ::GPDpr-ceHRG-4-HA-URA3 hap4Δ::HAP4-HA-KanMX4 pdr5Δ::NatMX4*	This study

Supplementary Table S2. Plasmids used in this study.

Plasmid	Description	Source
pPP111	pDZ415-HA	This study
pPP52	pRS306-ADH1pr	This study
pPP96	pYES-DEST52-ceHRG-4	Iqbal Hamza
pPP97	p416-GPD-ceHRG-4	This study

Supplementary Table S3. Oligonucleotides used in this study.

Oligonucleotides	Sequence	Purpose
oPP085_ACT1_F	TCGTTCCAATTTACGCTGGTT	RT-qPCR
oPP086_ACT1_R	CGGCCAAATCGATTCTCAA	RT-qPCR

oPP228_ACO1_F	GACCATTTTCACTGTTACTCC	RT-qPCR
oPP229_ACO1_R	GATATCTCTACGATCCCATTG	RT-qPCR
oPP238_KGD1_F	GATAAGAGGTTCGGTTTAGAAG	RT-qPCR
oPP239_KGD1_R	GTTTACGGACCACATTGGAT	RT-qPCR
oPP260_HAP4_F	CTGATTCTCCAGCAGATTTC	RT-qPCR
oPP261_HAP4_R	CGTTATTCGTGTTGACTTTG	RT-qPCR
oPP268_Hap4pr_F	TCTCCTAGTACATCAAAGAGC	*HAP4* deletion
oPP269_Hap4orf_R	TAAAATGGTTACTACGAGGGC	*HAP4* deletion
oPP291_SDH1_F	AGAGGTGTTGGTAAGAAAAAG	RT-qPCR
oPP292_SDH1_R	GGGAATACCACCCATGTTAT	RT-qPCR
oPP378_TEFpr_R	CTGCAGCGAGGAGCCGTAAT	ceHRG-4 sequencing
oPP399_HMX1pr_Ura3pr_F	ACAGCATATATACACACACACACATAAAATAACCGCAAAAttcaattcatcatttttttt	*HMX1* overexpression
oPP400_HMX1orf_ADHpr_R	CAGTGGGTGAGGGTATGATTGTATTGCTACTGTCCTCCATagttgattgtatgcttggta	*HMX1* overexpression
oPP431_URA3_GAPpr_F	AAGGATAAGTTTTGACCATCAAAGAAGGTTAATGTGGCTGtcattatcaatactcgccat	ceHRG-4 integration
oPP432_URA3_p416_R	TTGGATAGTTCCTTTTTATAAAGGCCATGAAGCTTTTTCTtactgagagtgcaccatacc	ceHRG-4 integration
oPP437_Hap4_HA_F	CCTTGACGAAGATGTCGATTTTTTAAAGGTACAAGTATTTagcttaggtggaatgtaccc	Hap4-HA integration
oPP438_Hap4_HA_R	TTTTTAGTTGTTTTCGTTTTATTGCAACATGCCTATTTCAgcataggccactagtggatc	Hap4-HA integration
oPP511_PDRF5_Tef_F	AAGTTTTCGTATCCGCTCGTTCGAAAGACTTTAGACAAAAgacatggaggcccagaatac	*PDR5* deletion
oPP512_PDRF5_Tef_R	TCTTGGTAAGTTTCTTTTCTTAACCAAATTCAAAATTCTAcagtatagcgaccagcattc	*PDR5* deletion
oPP274_Hap4pr_Ura3pr_F	CCTACATTTTCTAGTACAAAAAAAAAACAAAAAAAGAATCTTCAATTCATCATTTTTTTT	*HAP4* overexpression
oPP275_Hap4orf_ADHpr_R	GACTAGCGGAGGCCTGTAGTAGAAAAGTCTTTGCGGTCATAGTTGATTGTATGCTTGGTA	*HAP4* overexpression
